# Effect of Patient Sex on the Severity of Coronary Artery Disease in Patients with Newly Diagnosis of Obstructive Sleep Apnoea Admitted by an Acute Coronary Syndrome

**DOI:** 10.1371/journal.pone.0159207

**Published:** 2016-07-14

**Authors:** Alicia Sánchez-de-la-Torre, Jorge Abad, Joaquín Durán-Cantolla, Olga Mediano, Valentín Cabriada, María José Masdeu, Joaquín Terán, Juan Fernando Masa, Mónica de la Peña, Albina Aldomá, Fernando Worner, Joan Valls, Ferran Barbé, Manuel Sánchez-de-la-Torre, Spanish Sleep Group

**Affiliations:** 1 Respiratory Department. Group of Translational Research in Respiratory Medicine. Hospital Universitari Arnau de Vilanova and Santa Maria. IRBLleida. Lleida, Catalonia, Spain; 2 Centro de Investigación Biomédica en Red de Enfermedades Respiratorias (CIBERES). Madrid, Spain; 3 Respiratory Department, Hosp Universitari Germans Trias I Pujol, Badalona, Barcelona, Catalonia, Spain; 4 Bio-Araba Research Institute, Araba University Hospital. Department of Medicine of Basque Country University, Vitoria-Gasteiz Spain; 5 Respiratory Department, Hospital Universitario de Guadalajara. Guadalajara, Spain; 6 Respiratory Department, Hospital Universitario Cruces. Bilbao, Spain; 7 Respiratory Department, Hospital Parc Tauli. Sabadell, Barcelona, Catalonia. Spain; 8 Respiratory Department, Hospital General Yagüe. Burgos, Spain; 9 Respiratory Department, Hospital San Pedro Alcántara, Cáceres, Spain; 10 Clinic Analysis and Respiratory Services, Hospital Universitari Son Espases, Institut de investigació sanitaria de Palma (IdisPa), Palma de Mallorca, Spain; 11 Cardiology Department, Hospital Universitari Arnau de Vilanova. IRBLleida, Lleida, Catalonia, Spain; 12 Department of Statistics. IRB Lleida, Lleida, Catalonia, Spain; University Heart Center Freiburg, GERMANY

## Abstract

**Background:**

The cardiovascular consequences of obstructive sleep apnoea (OSA) differ by sex. We hypothesized that sex influences the severity of acute coronary syndrome (ACS) in patients with OSA. OSA was defined as an apnoea–hypopnoea index (AHI)>15 events·h^-1^. We evaluated the severity of ACS according to the ejection fraction, Killip class, number of diseased vessels, number of stents implanted and plasma peak troponin level.

**Methods:**

We included 663 men (mean±SD, AHI 37±18 events·h-^1^) and 133 women (AHI 35±18 events·h-^1^) with OSA.

**Results:**

The men were younger than the women (59±11 versus 66±11 years, p<0.0001), exhibited a higher neck circumference (p<0.0001), and were more likely to be smokers and alcohol users than women (p<0.0001, p = 0.0005, respectively). Body mass index and percentage of hypertensive patients or diabetics were similar between sexes. We observed a slight tendency for a higher Killip classification in women, although it was not statistically significant (p = 0.055). For men, we observed that the number of diseased vessels and the number of stents implanted were higher (p = 0.02, p = 0.001, respectively), and a decrease in the ejection fraction (p = 0.002).

**Conclusions:**

This study shows that sex in OSA influences the severity of ACS. Men show a lower ejection fraction and an increased number of diseased vessels and number of stents implanted.

## Introduction

Obstructive sleep apnoea (OSA) is a common disease that affects approximately 10% of the middle-aged population and becomes more prevalent with age. It has been estimated that OSA affects at least 17% of men and 9% of women [1,2]. Obstructive apnoeic events incorporate a range of stressors that activate mechanisms contributing to the initiation and progression of cardiac diseases [3]. According to published data, OSA has been associated with significant cardiovascular morbidity and mortality and seems to be an independent risk factor for cardiovascular diseases [4,5]. Epidemiological studies suggest an association between OSA and hypertension, coronary heart disease, heart failure, and stroke [4]. Moreover, OSA is associated with an increased severity of acute coronary syndrome (ACS) [6]. Nevertheless, the cardiovascular consequences of OSA differ by sex as epidemiological studies suggest sex-based differences in the association between OSA and cardiovascular outcomes [7–9]. The effect of the gender into OSA associated cardiovascular comorbidities remain incompletely understood because of the underrepresentation of women in prior studies exploring the burden of comorbidities associated with OSA. A recent study which includes a large sample size of men and women with OSA reported sex-differences on the prevalence of comorbidities associated with OSA, as ischaemic heart disease were more prevalent in men with OSA [10]. Further clinical trials are needed to assess the role of sex in the context of severity of cardiovascular comorbidities associated with OSA.

We hypothesized that sex-specific differences in the pathophysiology of OSA would result in significant sex differences in the severity of ACS. Exploring the sex-specific aspects of OSA and cardiovascular disease may have direct implications for risk stratification and management of these patients.

## Methods

### Study Design and Subjects

**Fig 1:**
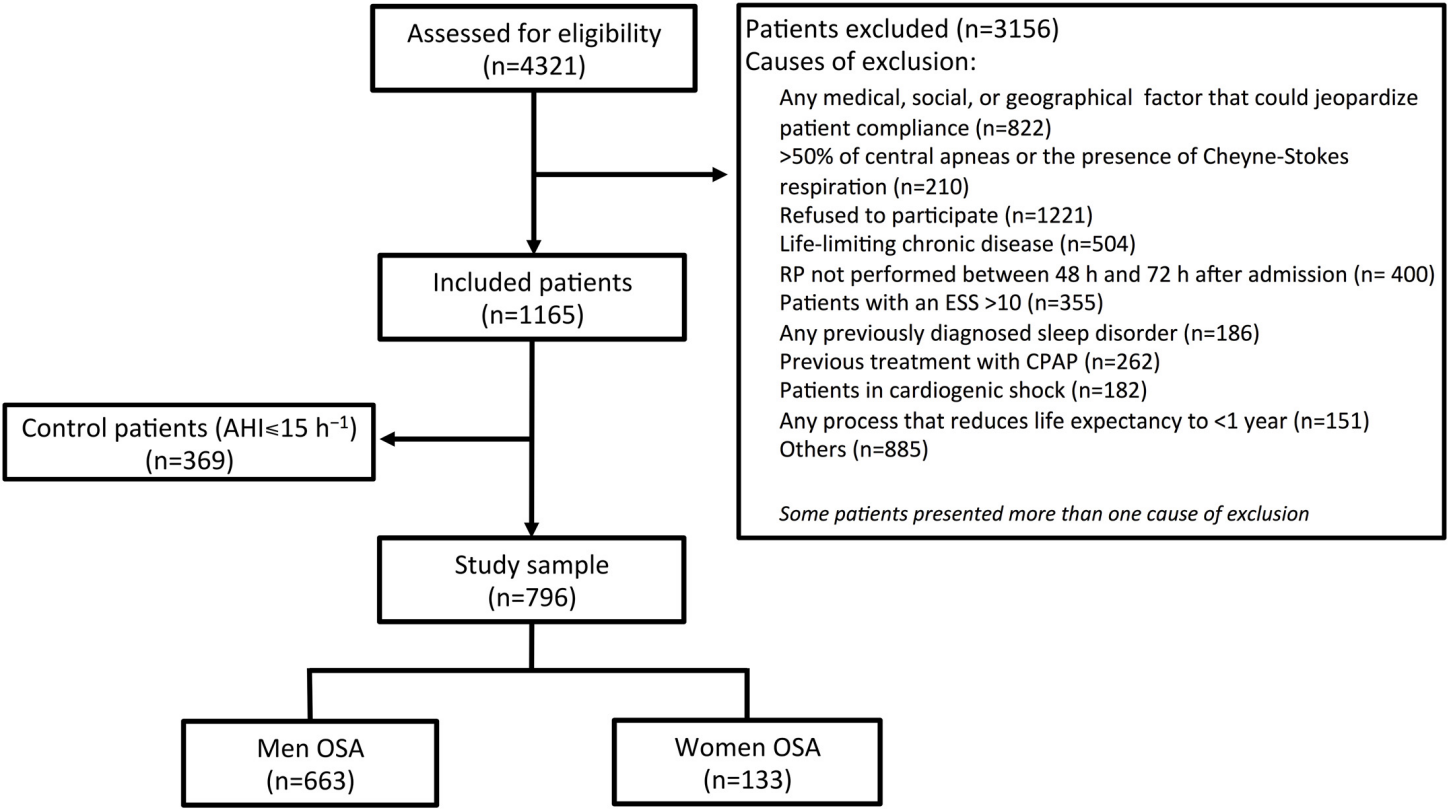
Study flowchart showing recruitment for the study. RP: respiratory polygraphy; ESS: Epworth Sleepiness Scale; CPAP: continuous positive airway pressure; OSA: obstructive sleep apnoea.

This was an ancillary study of the ISAACC study, a multicentre, open-label, parallel, prospective, randomized controlled trial (NCT01335087) (Continuous Positive Airway Pressure (CPAP) in Patients With Acute Coronary Syndrome and Obstructive Sleep Apnoea (ISAACC)) [11]. The ISAACC study assesses the impact of CPAP treatment on the incidence of new cardiovascular events in patients with ACS and OSA. Briefly, the ISAACC study include consecutive patients with an ACS diagnosis evaluated in coronary care units or cardiology hospitalization wards at fourteen participating hospitals in Spain (men and women aged ≥18 years). During a hospital stay, these patients are subjected to cardio-respiratory polygraphy. Patients with an apnoea–hypopnoea index (AHI) >15 events·h^−1^ are randomized to CPAP treatment or conservative treatment. Those patients with an AHI ≤15 events·h^−1^ are considered controls. In this study, we investigate the effects of gender on the severity of ACS in patients with OSA (Fig 1).

The exclusion criteria included previous treatment with CPAP; psychophysical inability to complete questionnaires; the presence of any previously diagnosed sleep disorder; patients with >50% central apnoeas or the presence of Cheyne–Stokes respiration; daytime sleepiness (Epworth Sleepiness Scale (ESS) >10); patients with chronic diseases, e.g., neoplasms, renal insufficiency (GFR <15 mL·min^-1^·1.73 m^−2^), severe chronic obstructive pulmonary disorder (a forced expiratory volume in 1 s <50%), chronic depression, and other limiting chronic diseases; a medical history that could interfere with the study objectives; any processes, whether cardiovascular or otherwise, that reduce life expectancy to <1 year; and patients in cardiogenic shock.

This study was approved by the ethics committee of each participating centre (approval number in the coordinator centre: “*Ethical Clinical Research Committee of Lleida (CEIC-Lleida)*”(2010–852)). All participants provided informed signed consent to participate in the study. This study was conducted according to the principles expressed in the Declaration of Helsinki.

### Procedures

The ISAACC study procedures have been described previously [6,11]. All the included patients underwent an attended respiratory polygraphy in the sleep laboratory of each centre, according to the guidelines of the Spanish national consensus on the apnoea-hypopnoea syndrome [12]. All the participating hospitals used the same model of polygraph (Embletta; ResMed, Bella Vista, Australia). Respiratory polygraphy included continuous recording of oronasal flow and pressure, heart rate, thoracic and abdominal respiratory movements, and oxygen saturation (SaO_2_). Polygraphy data were scored manually by trained personnel. Apnoea was defined as an interruption of oronasal airflow for more than 10 seconds. Hypopnoea was defined as a decrease in arterial oxygen saturation >4%. Respiratory polygraphy studies were performed without supplemental oxygen. The AHI was defined as the number of apnoeas plus hypopnoeas per hour of recording, and the TSat_90_ was defined as the percentage of recording time with a SaO_2_ less than 90%. The degree of daytime sleepiness (ESS score) was assessed in patients treated in the coronary care unit with a diagnosis of ACS. During patient admission, an electrocardiogram evaluation and Killip classification were performed. To provide a clinical estimate of the severity of the myocardial derangement, each patient was classified into one of the following classes: I) no heart failure, with no clinical signs of cardiac decompensation; II) heart failure as demonstrated by the presence of rales, an S3 gallop, and/or venous hypertension; III) severe heart failure or frank pulmonary oedema; and IV) cardiogenic shock [13]. During the patient´s hospital stay, we evaluated the severity of ACS according to the ejection fraction, the Killip scale, number of affected vessels, number of stents implanted and peak troponin level.

### Statistical analysis

Mean (and standard deviation) or absolute frequency (and percentage) was used to describe quantitative and qualitative variables, respectively. First, a homogeneity analysis was performed to compare men and women with regards to anthropometric, clinical, treatment variables and ACS-related risk factors, using the Mann-Whitney test or Fisher test to assess the significance of the differences. Second, the association of sex with ACS severity-related variables was assessed using linear regression models and computing the corresponding p-values. Variables potentially confounded with sex, as detected in the homogeneity analysis, were also considered in the models to calculate an adjusted p-value (age, ESS, neck circumference, tobacco exposure (pack-years) and alcohol). Finally, an analysis to evaluate the association of sex with ACS severity variables, while taking into account the OSA severity (moderate OSA for AHI between 15–30 vs. severe OSA for AHI ≥30), was performed. For this purpose, stratified analyses for moderate and severe OSA patients were performed, using the same models as previously described. Further homogeneity analyses to detect variables potentially confounded to sex were also applied by each OSA severity level to determine the variables to be considered in the adjusted models. All analyses were performed using the R statistical package, setting the threshold for significance at 5% (α = 0.05).

### Sample size

The sample population included 133 women and 663 men, which provided a statistical power of 80% to detect a minimum increase in mean troponin levels (scaled) of 16.25% and a minimum decrease in the mean ejection fraction of 5.09% in men with respect to women. These calculations were performed based on the mean values and standard deviations reported in Table 1 and using a two-sample T-test to statistically assess the differences with a 5% type I error (α = 0.05).

**Table 1:** Differences in ACS severity-related variables between men and women with OSA.

	OSA patients (n = 796)	Sex		p-value	Adjusted p-value
		Women—(n = 133)	Men—(n = 663)		
ACS category *				0.34	0.8
Unstable	74 (11.56%)	17 (16.50%)	57 (10.61%)		
Non-STEMI	306 (47.81%)	45 (43.69%)	261 (48.60%)		
STEMI	260 (40.62%)	41 (39.81%)	219 (40.78%)		
Killip classification *				**0.02**	0.055
Killip I	544 (89.92%)	86 (84.31%)	458 (91.05%)		
Killip II	52 (8.60%)	12 (11.76%)	40 (7.95%)		
Killip III	6 (0.99%)	3 (2.94%)	3 (0.60%)		
Killip IV	3 (0.50%)	1 (0.98%)	2 (0.40%)		
Number of diseased vessels *				**0.02**	**0.02**
0	17 (2.41%)	5 (4.31%)	12 (2.04%)		
1	333 (47.30%)	61 (52.59%)	272 (46.26%)		
2	198 (28.12%)	33 (28.45%)	165 (28.06%)		
3	156 (22.16%)	17 (14.66%)	139 (23.64%)		
Number of stents implanted	1.47 (0.99)	1.19 (0.68)	1.53 (1.03)	**0.001**	**0.001**
Ejection fraction, %	55.02 (10.83)	57.38 (11.16)	54.54 (10.71)	**0.02**	**0.002**
Peak troponin, rank^+^	0.50 (0.29)	0.48 (0.30)	0.51 (0.29)	0.48	0.11
CPK, U/l	744.45 (1133.58)	582.24 (879.00)	776.70 (1175.71)	0.12	0.17

Data are presented as the mean (and standard deviation) and frequency (and percentage) for quantitative and qualitative variables, respectively. P-values and adjusted p-values to assess the differences were computed with linear regression models, considering age, ESS, neck circumference, tobacco exposure (pack-years) and alcohol for adjustment.

* Numerical order for the categories was considered in the linear regression models.

^+^ For assessing differences in the peak troponin levels, the rank of the data was used for each type of troponin evaluated.

## Results

A total of 663 men and 133 women with OSA were included. Anthropometric, clinical variables and ACS-related risk factors are presented in Table 2. The women were older than the men (p<0.0001), and the men showed slightly, but significantly, higher ESS scores than the women (p = 0.03). Additionally, men exhibited a higher neck circumference (p<0.0001) and were more likely than women to be smokers and alcohol users (p<0.0001, p = 0.0005, respectively). Compared with men, women used more diuretics and antacids (p = 0.0006, p = 0.0008, respectively).

**Table 2:** Differences in anthropometric, clinical, and treatment variables and ACS-related risk factors between men and women with OSA.

	OSA patients (n = 796)	Sex		p-value
		Women (n = 133)	Men (n = 663)	
Age, years	60.46 (10.49)	65.56 (10.51)	59.44 (10.19)	**<0.00001**
Apnoea-hypopnoea index events·h^-1^	36.48 (18.09)	35.04 (17.70)	36.77 (18.17)	0.27
Oxigen desaturation endex >4% h^-1^	34.22 (37.23)	30.98 (37.79)	34.90 (37.11)	0.1
Minimum SaO_2_, %	80.87 (10.10)	81.25 (10.21)	80.79 (10.08)	0.76
Mean SaO_2_, %	90.74 (12.60)	91.11 (11.60)	90.66 (12.80)	0.8
Time with SaO_2_ <90%, min	51.48 (82.09)	57.56 (88.91)	50.24 (80.64)	0.98
Time with SaO_2_ <90%, %	14.09 (39.44)	14.23 (20.36)	14.06 (42.26)	0.76
Epworth Sleepiness Scale	5.46 (2.49)	4.99 (2.59)	5.55 (2.46)	**0.03**
Hypertensive patients	406 (53.99%)	76 (60.32%)	330 (52.72%)	0.14
Body mass index, kg·m^-2^	29.36 (4.91)	29.82 (5.45)	29.27 (4.80)	0.67
Neck circumference, cm	41.17 (3.53)	37.59 (3.04)	41.86 (3.18)	**<0.00001**
Diabetes mellitus	184 (24.50%)	34 (26.98%)	150 (24.00%)	0.5
Dyslipidaemia	392 (52.06%)	73 (57.94%)	319 (50.88%)	0.17
First episode of ACS	569 (82.11%)	91 (81.98%)	478 (82.13%)	1
Cardiomyopathy	163 (21.97%)	28 (22.76%)	135 (21.81%)	0.81
Stroke	25 (3.39%)	1 (0.82%)	24 (3.90%)	0.1
Current or former smoker	543 (72.40%)	59 (47.58%)	484 (77.32%)	**<0.00001**
Smoker				**0.0005**
No	207 (27.60%)	65 (52.42%)	142 (22.68%)	
Yes	348 (46.40%)	42 (33.87%)	306 (48.88%)	
Former smoker	195 (26.00%)	17 (13.71%)	178 (28.43%)	
Total tobacco exposure, pack-years	23.19 (27.30)	13.64 (21.21)	25.32 (28.05)	**<0.00001**
Alcohol				**0.0005**
No	521 (72.66%)	116 (95.87%)	405 (67.95%)	
Yes	190 (26.50%)	5 (4.13%)	185 (31.04%)	
Former alcoholism	6 (0.84%)	0 (0%)	6 (1.01%)	
Diuretics	141 (19.13%)	38 (31.15%)	103 (16.75%)	**0.0006**
Anticoagulants	51 (6.93%)	11 (9.02%)	40 (6.51%)	0.33
Antacids	202 (27.48%)	49 (40.16%)	153 (24.96%)	**0.0008**
Hypolipidemics	274 (37.18%)	52 (42.62%)	222 (36.10%)	0.18
β-Blockers	160 (21.71%)	30 (24.59%)	130 (21.14%)	0.4
Calcium antagonists	108 (14.69%)	22 (18.03%)	86 (14.03%)	0.26
Antiplatelet	165 (22.45%)	30 (24.59%)	135 (22.02%)	0.55
Insulin	48 (6.53%)	13 (10.66%)	35 (5.71%)	0.07
Oral antidiabetics	140 (19.02%)	26 (21.31%)	114 (18.57%)	0.53

Data are presented as the mean (and standard deviation) and absolute frequency (and percentage) for quantitative and qualitative variables, respectively. SaO_2_: arterial oxygen saturation. P-values to assess differences in means or proportions between groups were computed using Mann-Whitney and Fisher tests, respectively.

**Fig 2:**
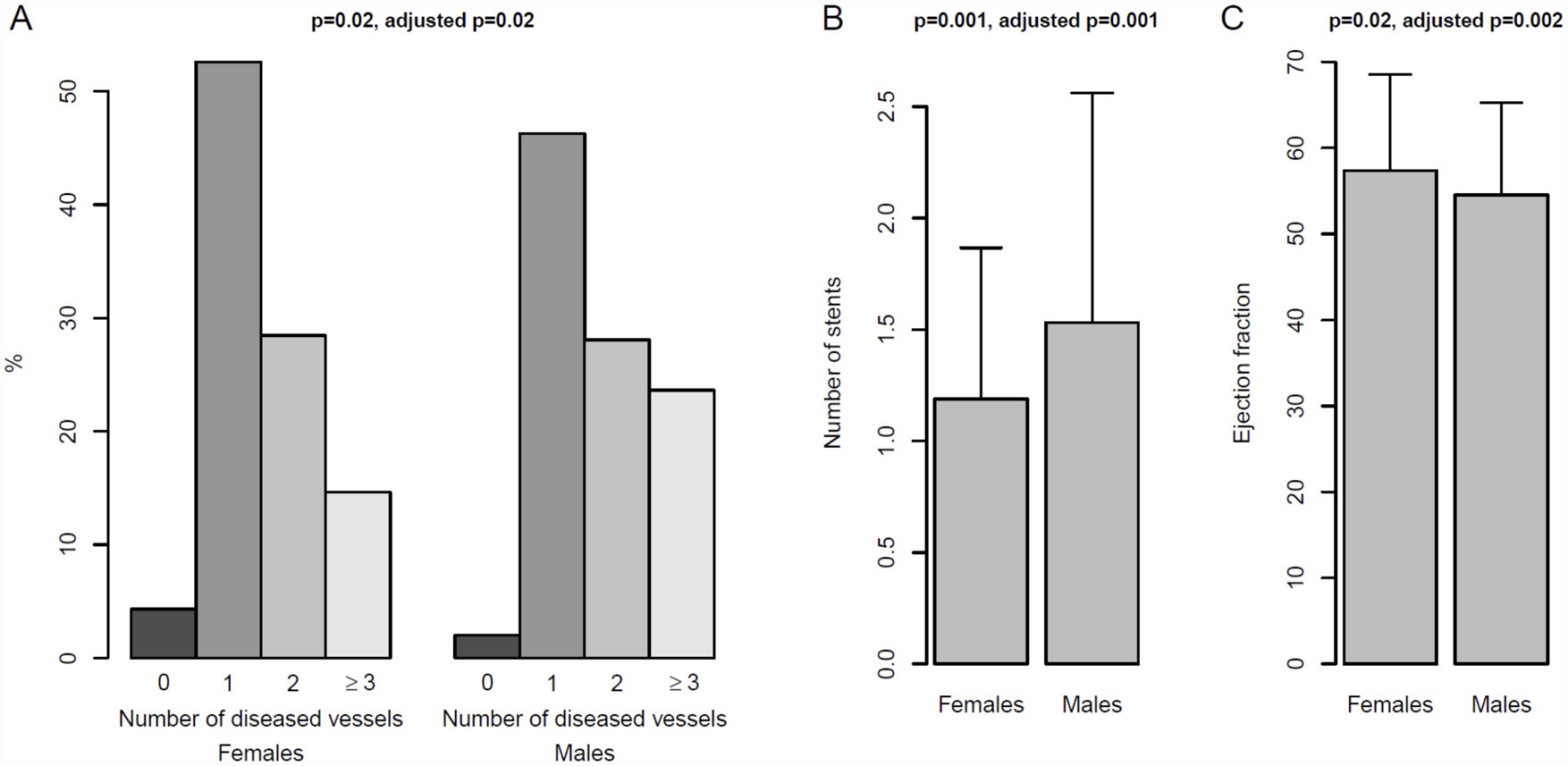
Number of diseased vessels (A), number of stents (B) and ejection fraction (C) in men and women with OSA. Percentages and means are represented by bars, and the standard deviation is represented by segments. P-values from linear regression models to assess the significance of the association are shown. Adjusted p-values including total tobacco exposure (pack-years), age, Epworth sleep score, neck circumference and alcohol are also shown. Integer values were considered for models in (A).

In relation to ACS severity, the percentages of patients with Killip classes II-IV were higher among women (p = 0.02). The analysis adjusted for age, ESS score, neck circumference, tobacco exposure (pack-years) and alcohol revealed a slight tendency for a higher Killip classification in women, although this difference was not statistically significant (p = 0.055). Mean troponin levels were similar between groups (p = 0.48) (Table 1). For the group of men, the adjusted analysis showed that the number of diseased vessels and number of stents implanted were higher (p = 0.02, p = 0.001, respectively), and the men also exhibited a decrease in the ejection fraction (p = 0.002) (Table 1) (Fig 2).

**Fig 3:**
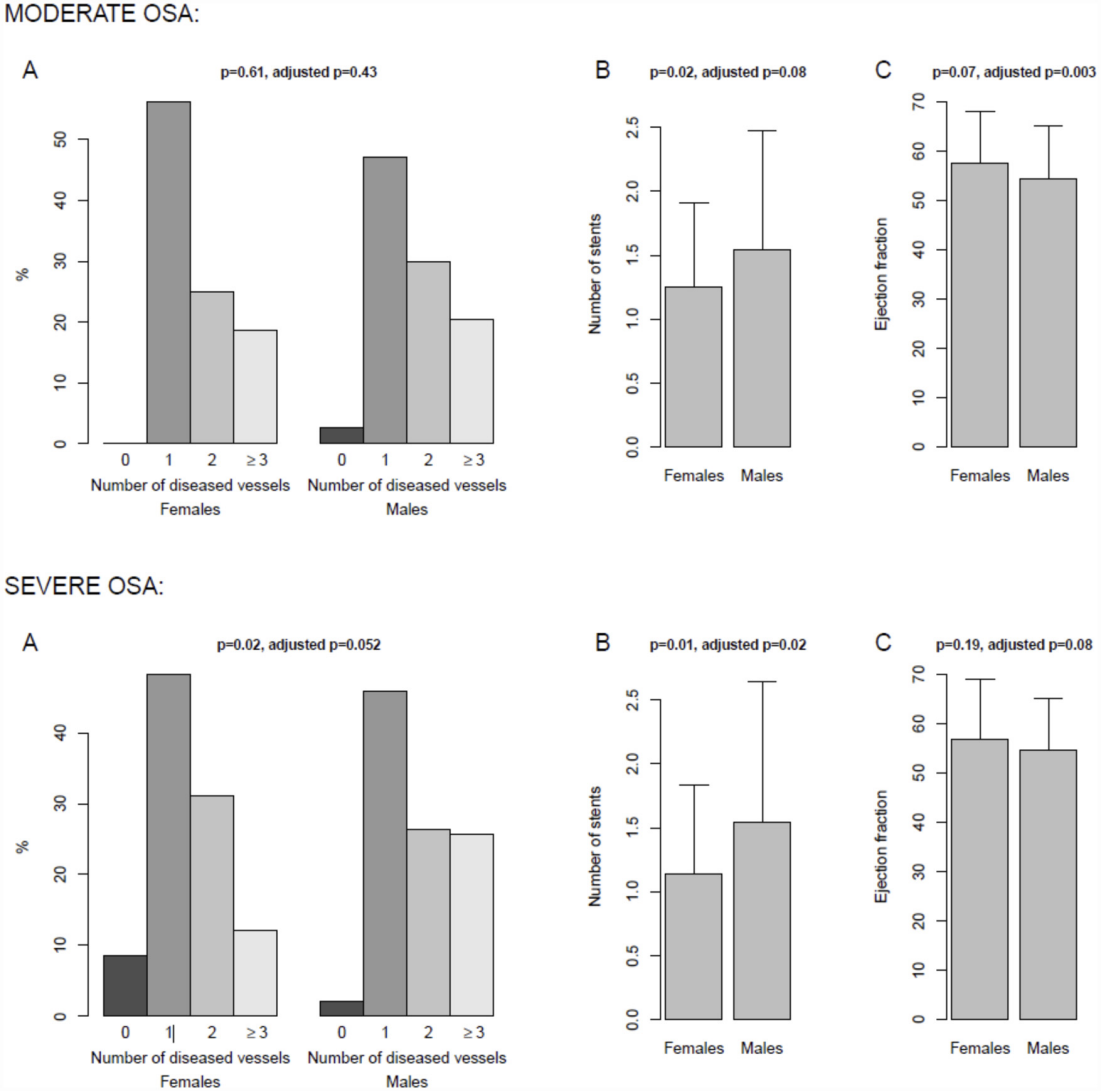
Number of diseased vessels (A), number of stents (B) and ejection fraction (C) in men and women with moderate or severe OSA. Percentages and means are represented by bars, and the standard deviation is represented by segments. P-values from linear regression models to assess the significance of the association are shown. Adjusted p-values including total tobacco exposure (pack-years), age, Epworth sleep score, neck circumference and alcohol are also shown. Integer values were considered for models in (A).

To further evaluate the strength of the effect of the sex of OSA patients on ACS severity-related variables, we classified the patients into two groups according to OSA severity; specifically, we analysed the differences in sex for ACS severity-related variables in patients with severe (AHI ≥30 events·h^-1^) and moderate (AHI 15–30 events·h^-1^) OSA. The results showed that men with moderate OSA had a higher number of stents implanted (p = 0.02) than women with moderate OSA. Moreover, the analysis adjusted by age, ESS score, neck circumference, dyslipidemia, tobacco exposure and alcohol revealed that the mean ejection fraction was lower in men with moderate OSA (p = 0.003). For severe OSA patients, the men exhibited a lower Killip classification, a higher number of diseased vessels and a higher number of stents implanted (p = 0.001, p = 0.02 and p = 0.01, respectively). Nevertheless, after adjustment, only the number of stents implanted was statistically significant (p = 0.02) (Table 3) (Fig 3).

**Table 3:** Differences in ACS severity-related variables between men and women with moderate or severe OSA.

	Moderate OSA (AHI 15–30)					Severe OSA (AHI≥30)				
	OSA patients (n = 377)	Sex		p-value	Adjusted p-value (1)	OSA patients (n = 446)	Sex		p-value	Adjusted p-value (2)
		Women (n = 72)	Men (n = 305)				Women (n = 67)	Men (n = 379)		
ACS category *				0.58	0.38				0.45	0.64
Unstable	37 (12.59%)	9 (15.52%)	28 (11.86%)			42 (11.29%)	9 (17.65%)	33 (10.28%)		
Non-STEMI	136 (46.26%)	26 (44.83%)	110 (46.61%)			176 (47.31%)	21 (41.18%)	155 (48.29%)		
STEMI	121 (41.16%)	23 (39.66%)	98 (41.53%)			154 (41.40%)	21 (41.18%)	133 (41.43%)		
Killip classification *				0.82	0.33				**0.001**	0.16
Killip I	261 (91.9%)	49 (90.74%)	212 (92.17%)			300 (87.72%)	40 (75.47%)	260 (89.97%)		
Killip II	22 (7.75%)	5 (9.26%)	17 (7.39%)			34 (9.94%)	9 (16.98%)	25 (8.65%)		
Killip III	1 (0.35%)	0 (0%)	1 (0.43%)			5 (1.46%)	3 (5.66%)	2 (0.69%)		
Killip IV	0 (0%)	0 (0%)	0 (0%)			3 (0.88%)	1 (1.89%)	2 (0.69%)		
Number of diseased vessels *				0.61	0.43				**0.02**	0.052
0	7 (2.07%)	0 (0%)	7 (2.55%)			12 (3.08%)	5 (8.62%)	7 (2.11%)		
1	165 (48.82%)	36 (56.25%)	129 (47.08%)			180 (46.27%)	28 (48.28%)	152 (45.92%)		
2	98 (28.99%)	16 (25.00%)	82 (29.93%)			105 (26.99%)	18 (31.03%)	87 (26.28%)		
3	68 (20.12%)	12 (18.75%)	56 (20.44%)			92 (23.65%)	7 (12.07%)	85 (25.68%)		
Number of stents implanted	1.48 (0.89)	1.25 (0.65)	1.54 (0.93)	**0.02**	0.08	1.48 (1.06)	1.14 (0.69)	1.54 (1.10)	**0.01**	**0.02**
Ejection fraction, %	54.97 (10.76)	57.53 (10.39)	54.35 (10.78)	0.07	**0.003**	54.88 (10.86)	56.81 (12.12)	54.54 (10.61)	0.19	0.08
Peak troponin, rank^+^	0.50 (0.29)	0.48 (0.28)	0.51 (0.29)	0.4	0.33	0.50 (0.29)	0.50 (0.32)	0.50 (0.28)	0.97	0.24
CPK, U/l	705.99 (1103.03)	573.05 (886.65)	739.23 (1,150.05)	0.31	0.85	764.01 (1,152.41)	551.33 (842.10)	798.61 (1,192.87)	0.17	0.08

Data are presented as the mean (and standard deviation) and absolute frequency (and percentage) for quantitative and qualitative variables, respectively. P-values and adjusted p-values to assess the differences were computed with linear regression models, considering (1) age, ESS score, neck circumference, dyslipidaemia, tobacco exposure (pack-years) and alcohol for adjustment in moderate OSA patients and (2) age, neck circumference, tobacco exposure (pack-years), alcohol, diuretics, antacids and insulin for adjustment in severe OSA patients, respectively.

* Numerical order for the categories was considered in the linear regression models.

^+^ For assessing differences in the peak troponin levels, the rank of the data was used for each type of troponin evaluated

## Discussion

The results of this observational study suggest that in patients with OSA, sex influences the severity of ACS. Men with OSA showed a higher ACS severity than women, as well as a decrease in the ejection fraction, and an increase in the number of diseased vessels and the number of stents implanted. To further investigate the strength of the association of ACS severity-related variables with OSA severity, we classified male and female patients according to OSA severity. These results showed that with increasing severity of OSA, the gender-related differences in the severity of ACS were more evident as men with severe OSA were also those with a higher severity of ACS, with a higher number of stents implanted and a greater tendency to have a higher number of affected vessels than women with severe OSA. Cigarettes and alcohol are well known risk factors for OSA and cardiovascular disease. The fact that men were significantly more exposed to both of these risk factors could explain the differences in severity. Nevertheless, the results previously indicated were adequately adjusted for this potentially confounding variables.

Despite several confounding factors, including age, sex and obesity, there is accumulating evidence regarding strong associations between OSA and cardiovascular diseases, such as hypertension [14,15], coronary artery disease (CAD) [16] and cerebrovascular disease [17–19]. Although the coexistence of these conditions with OSA not prove causality, epidemiological data support the concept that OSA can participate in the initiation or progression of several cardiovascular diseases [20–26]. Previous studies have suggested that sex influences the cardiovascular risk in OSA. However, the limited number of studies addressing the impact of sex on the relationship between OSA and cardiovascular outcomes have yielded conflicting results. The Sleep and Heart Health Study showed that men, but not women, with severe OSA showed higher rates of total mortality [8] and cardiovascular events [7]. This study did not find any association between OSA and mortality or other incident cardiovascular outcomes, including stroke and coronary heart disease, in 3,000 women followed for over 8 years. However, severe OSA accounted for only 3% of all the women sampled in the Sleep and Heart Health Study, which might have biased the results. In contrast, a recent observational study in a cohort of middle-aged (mean age 62.5±5.5 years) community-dwelling individuals comprising 752 men and 893 women followed for 13 years showed that OSA was associated with incident heart failure or death only among women [9]. Together, this suggests that although women seem more vulnerable to the cardiovascular consequences of OSA, the present study showed that men show a greater severity of ACS.

The result of this study suggest that men showed an increased vascular involvement (higher number of diseased vessels and number of stents implanted and lower ejection fraction). However, troponin levels related to ACS are similar between sex. A plausible explanation is that the increased structural damage at myocardial level in men does not have to be accompanied by greater myocardial damage when an acute ischemic episode occurs. Indeed, after and ACS the consequences could be similar between gender in part due to the ischemic preconditioning previously postulated [29]. The influence of sex on the severity of ACS in OSA that we observed in the present study may reflect sex differences in the pathobiology of OSA. In fact, the activation of several pathogenic factors proposed as intermediate mechanisms linking OSA with cardiovascular disease such us sympathetic activation [27] and endothelial dysfunction [27,28] varies between men and women. Moreover, OSA severity is more strongly positively associated with higher plasma levels of biomarkers of subclinical myocardial injury such us high-sensitivity troponin T in women compared with men [9]. The cardiac adaptations to haemodynamic stress could also differ by sex, and these differences could explain why women with OSA are better adapted to cardiac stress than men; this would also support the development of ischaemic preconditioning in women that has been postulated in OSA [29]. Therefore, the sex differences in the pathobiology of OSA may reflect differences in the response to cardiovascular stressors and/or differences in the influence of OSA on cardiac injury or other cardiovascular diseases.

The strength of our study was its multicentre design that comprised a large number of patients. All participating centres performed the same methodology, and the sleep study was performed with the same polygraph model. Nevertheless, our findings must be interpreted in light of the limitations of the present study. First, we excluded patients with daytime sleepiness that could be related to the most severe forms of OSA. However, the number of excluded patients for these causes was relatively low (8%). Second, the diagnosis of OSA was based on respiratory polygraphy, which could underestimate the severity of OSA. However, due to the critical situation of the patients, full polysomnography monitoring could be a stressful procedure for this high-risk patient group. Moreover, numerous studies have demonstrated the utility of respiratory polygraphy for OSA diagnosis with similar results to polysomnography. Third, this study comprises a limited number of women. However, this study is a large series of patients consecutively included that reflect the incidence of this disease, and moreover, sample size allows us to meet the objectives raised with an adequate statistical power. Despite these limitations, our study provides evidence that sex in OSA influences the severity of ACS.

## Conclusions

The results of the present study show that in OSA patients, patient sex influences the severity of ACS, such that men showed an increase in the number of diseased vessels and number of stents implanted and a lower ejection fraction with respect to women. The results of this study indicate that personalized clinical attention in patients with OSA and ACS should be adequately adapted based on sex. Further research assessing the survival of male and female OSA patients with ACS in population-based studies is required before conclusions can be made regarding patient survival.
